# Visualization of Endogenous Type I TGF-β Receptor Baboon in the *Drosophila* Brain

**DOI:** 10.1038/s41598-020-61950-y

**Published:** 2020-03-20

**Authors:** Yen-Wei Lai, Sao-Yu Chu, Jian-Chiuan Li, Po-Lin Chen, Chun-Hong Chen, Hung-Hsiang Yu

**Affiliations:** 1 Institute of Cellular and Organismic Biology, Academia Sinica, Taipei, Taiwan; 2 National Institute of Infectious Diseases and Vaccinology, National Health Research Institutes, Miaoli County, Taiwan; 3 Institute of Molecular and Cellular Biology, College of Life Science, National Taiwan University, Taipei, Taiwan

**Keywords:** Biological metamorphosis, Neuronal development

## Abstract

The transforming growth factor β (TGF-β) signaling pathway is evolutionarily conserved and widely used in the animal kingdom to regulate diverse developmental processes. Prior studies have shown that Baboon (Babo), a *Drosophila* type I TGF-β receptor, plays essential roles in brain development and neural circuit formation. However, the expression pattern for Babo in the developing brain has not been previously reported. We generated a knock-in fly with a human influenza hemagglutinin (HA) tag at the C-terminus of Babo and assessed its localization. Babo::HA was primarily expressed in brain structures enriched with neurites, including the mushroom body lobe and neuropils of the optic lobe, where Babo has been shown to instruct neuronal morphogenesis. Since the *babo* 3' untranslated region contains a predicted microRNA-34 (miR-34) target sequence, we further tested whether Babo::HA expression was affected by modulating the level of miR-34. We found that Babo was upregulated by *mir-34* deletion and downregulated by miR-34 overexpression, confirming that it is indeed a miR-34 target gene. Taken together, our results demonstrate that the *babo*^*HA*^ fly permits accurate visualization of endogenous Babo expression during brain development and the construction of functional neural circuits.

## Introduction

The transforming growth factor β (TGF-β) signaling pathway is evolutionarily conserved and widely utilized in animals as a regulator of diverse and complex processes, such as cell growth, differentiation and morphogenesis during development^1,2^. In *Drosophila*, two major groups of ligands, Activins and Bone morphogenetic proteins, stimulate distinct TGF-β signaling pathways that involve different sets of downstream effectors (reviewed by Upadhyay *et al*.^1^). Activin signals bind to Baboon (Babo), a type I TGF-β receptor that transduces TGF-β signaling by binding to type II TGF-β receptors, Punt and Wishful thinking (Wit), to regulate several essential processes in brain development and neural circuit formation^1,3–5^. For instance, Activin-β and an Activin-like protein Dawdle were shown to signal through Babo to regulate neuroblast proliferation in the larval brain, with loss of *babo* function resulting in fewer cells within the central brain and optic lobe^4^. Besides controlling neuroblast proliferation, Babo also transduces the signals of Activin-β and Activin-like protein Myoglianin to control neuronal morphogenesis during neural circuit formation, including in mushroom body (MB) neurons of the central brain and in medulla projection (Tm) neurons of the optic lobe^3,5,6^. When *babo* is mutated, MB neurons display both axon growth and pruning defects^3,7^, while Tm neurons exhibit multiple dendritic patterning phenotypes^5^. Taken together, these studies demonstrate the crucial role of Babo in brain development and in neural circuit formation.

Despite the fact that Babo is known to act as an important regulator, the Babo pattern expression in the brain has not been previously reported. In this study, we overcame a lack of reliable Babo antibodies by generating a *babo*^*HA*^ knock-in fly, which expresses a human influenza hemagglutinin (HA) tag at the C-terminus of the endogenous Babo protein. We then used immunostaining of the Babo::HA protein to delineate the expression pattern of endogenous Babo. Babo expression was observed in brain structures enriched with neurites, including nerve fibers and neuropils (e.g., MB and optic lobes). We then further utilized the *babo*^*HA*^ knock-in fly to address a previously unanswered question of whether *babo* is a true target gene for microRNA-34 (miR-34)^8,9^, finding that Babo expression was indeed regulated by miR-34. Taken together, this *babo*^*HA*^ fly serves as a powerful reagent to clarify the participation of Babo in the brain development and functional neural circuit formation.

## Results

### Commercially available Babo antibodies fail to faithfully detect Babo expression

**Figure 1:**
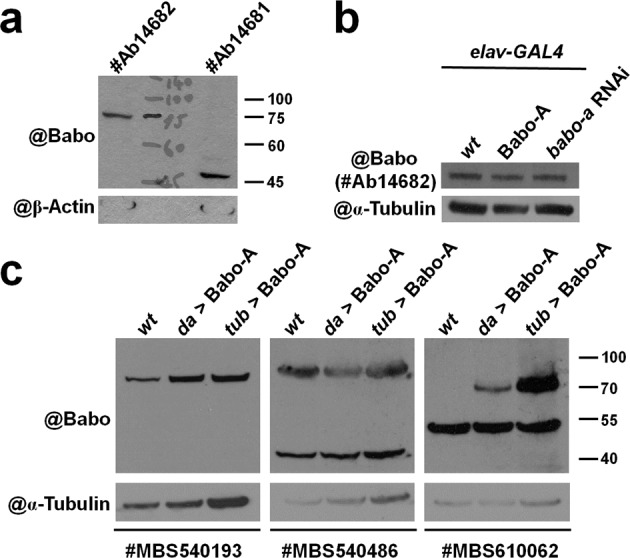
Commercial Babo antibodies cannot be used to faithfully detect Babo expression by western blot. (**a-c**) Five commercial Babo antibodies were characterized to determine whether any can faithfully detect Babo expression with a predicted size of 66–69 kD in western blot analyses. (**a**) Two Abcam antibodies (#Ab14681 and #Ab14682) were used to detect Babo expression in wild-type (*wt*) samples. (**b**) #Ab14682 was further used to probe the Babo expression in late third instar larval brain samples of *wt*, Babo-A-overexpressing and *babo-a* RNAi knockdown driven by a pan-neural GAL4 line, *elav-GAL4*. (**c**) Three additional MyBioSource antibodies (#MBS540193, #MBS540486 and #MBS610062) were also used to probe Babo expression in late third instar larval samples of *wt* and Babo-A-overexpressing lines driven by two pan-cell GAL4 lines, *da-GAL4* (weakly expressed) and *tub-GAL4* (strongly expressed). β-Actin and α-Tubulin were used as internal controls in Figs. 1, 2,4. Genotypes of flies shown in all figures are listed in Supplemental Table 1.

We first collected and characterized commercially available Babo antibodies with the hope that one could be used to track the expression of endogenous Babo during brain development. Two commercial antibodies (Abcam #14681 and #14682) were tested to determine if either can be used in a western blot to detect Babo protein isoforms with predicted molecular weights of 66–69 kD (Fig. 1a,b). The #14682 antibody detected a protein band at 75 kD, while the #14681 antibody did not recognize any protein near the 66–69 kD range (Fig. 1a), suggesting that of these two antibodies, only the #14682 antibody has potential to be a useful Babo antibody. However, we quickly ruled out the #14682 antibody as a useful Babo antibody when we found it could not faithfully detect increased or decreased Babo expression in the late third instar larval brain following overexpression or knockdown of Babo with the *elav-GAL4* pan-neural driver (Fig. 1b). We then evaluated three additional commercial antibodies (MyBioSource #540193, #540486 and #610062) by western blotting. Similar to the previous antibodies, these three also failed to detect either endogenous Babo expression (#610062 antibody; right panel of Fig. 1c) or increased Babo expression (#540193 and #540486 antibodies; left and middle panels of Fig. 1c) when Babo was overexpressed by two ubiquitously expressed drivers, *da-GAL4* (weakly expressed) and *tub-GAL4* (strongly expressed). Taken together, these results suggested that direct immunodetection of Babo may be a suboptimal strategy for tracking its endogenous expression in the brain.

### Visualization of endogenous Babo expression using a *babo*^*HA*^ knock-in fly

**Figure 2:**
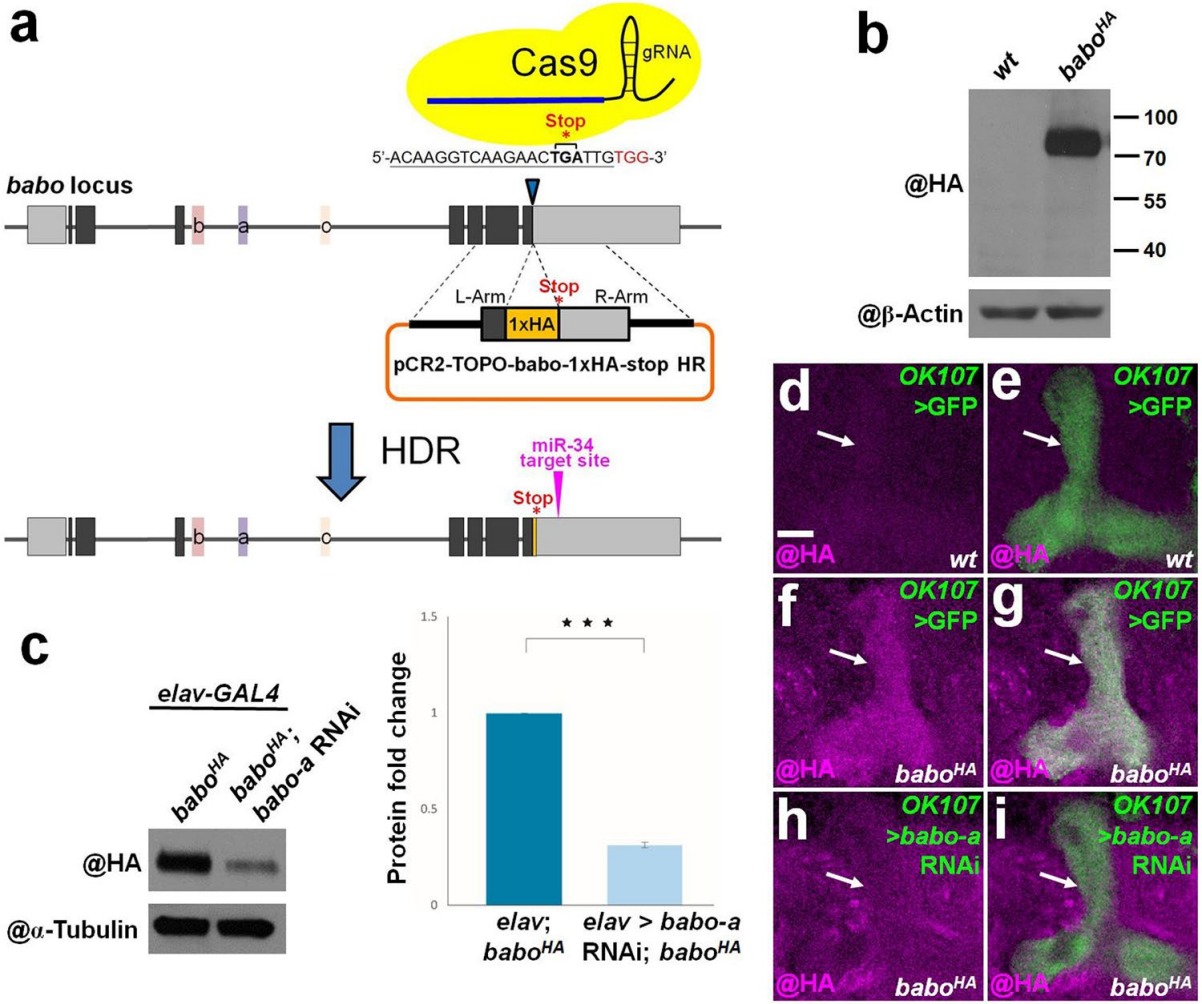
Generation of the *babo*^*HA*^ knock-in fly using CRISPR-Cas9 to reveal the endogenous Babo expression pattern. (**a**) A schematic illustrates the CRISPR-Ca9 strategy used to generate the *babo*^*HA*^ fly. In the gene diagram, three alternative splicing exons encoding sequences specific to different Babo protein isoforms are labeled as colored boxes with a, b and c. A putative miR-34 target site is indicated in the 3'untranslated region (UTR) of the *babo* gene. HR: homologous recombination; HDR: homology-directed repair. (**b**) A Babo::HA protein band around 70 kD was detected by western blot in the *babo*^*HA*^ but not the *wt* sample. (**c**) Babo::HA expression was significantly reduced in the late third instar larval brain when *babo-a* RNAi was overexpressed by *elav-GAL4*. (**d-i**) The specificity of Babo::HA was further validated in MB lobes of late third instar larval brain, using single confocal sections of *wt* (**d,e**), *babo*^*HA*^ (**f,g**), and *babo*^*HA*^ with *babo-a* RNAi driven by the *GAL4-OK107* pan-MB GAL4 line (**h,i**). HA staining (magenta) and GFP signal (green) show Babo::HA expression and the position of MB lobes, respectively. Scale bar: 10 μm for panels d-i.

Since we could not unambiguously detect Babo with commercially available antibodies, we used a CRISPR-Cas9-based strategy to generate a knock-in fly with a DNA fragment encoding the human influenza hemagglutinin (HA) tag inserted into the *babo* locus (Fig. 2a)^10,11^. We reasoned that this strategy would permit us to visualize the pattern of endogenous Babo expression by labeling Babo::HA with HA immunostaining. Based on previous studies and Flybase annotation, the *babo* gene is known to encode three Babo isoforms, Babo-A, Babo-B and Babo-C^12,13^, which all have the same C-terminus (Fig. 2a). Therefore, we generated a *babo*^*HA*^ fly by knocking-in the HA tag at the C-terminus of *babo*, immediately preceding the translational stop codon (Fig. 2a). We then inspected whether the *babo*^*HA*^ fly can be used to faithfully detect endogenous Babo expression. First, we detected Babo::HA expression in the *babo*^*HA*^ fly but not in the wild-type *w*^1118^ (*wt*) fly by western blot (Fig. 2b). We further found that Babo::HA expression level was significantly reduced in the late third instar larval brain homogenates when we overexpressed a *babo-a* RNA interference (*babo-a* RNAi) driven by *elav-GAL4* (31 ± 2%, *p* < 0.001; Fig. 2c). Next, we overexpressed the same *babo-a* RNAi under the control of the *GAL4-OK107* pan-MB driver to block the Babo-A expression in MB neurons^6^. According to our immunostaining results, Babo::HA expression in the MB lobe was greatly reduced when *babo-a* RNAi was overexpressed in MB neurons (Fig. 2d–i), consistent with the known role of Babo-A in regulating axon pruning of MB neurons^6^. Taken together, these results validate the utility of the *babo*^*HA*^ fly and suggest that its localization faithfully reflects endogenous Babo expression patterns.

### Babo::HA is primarily expressed in neuropils and nerve fibers of the brain

**Figure 3:**
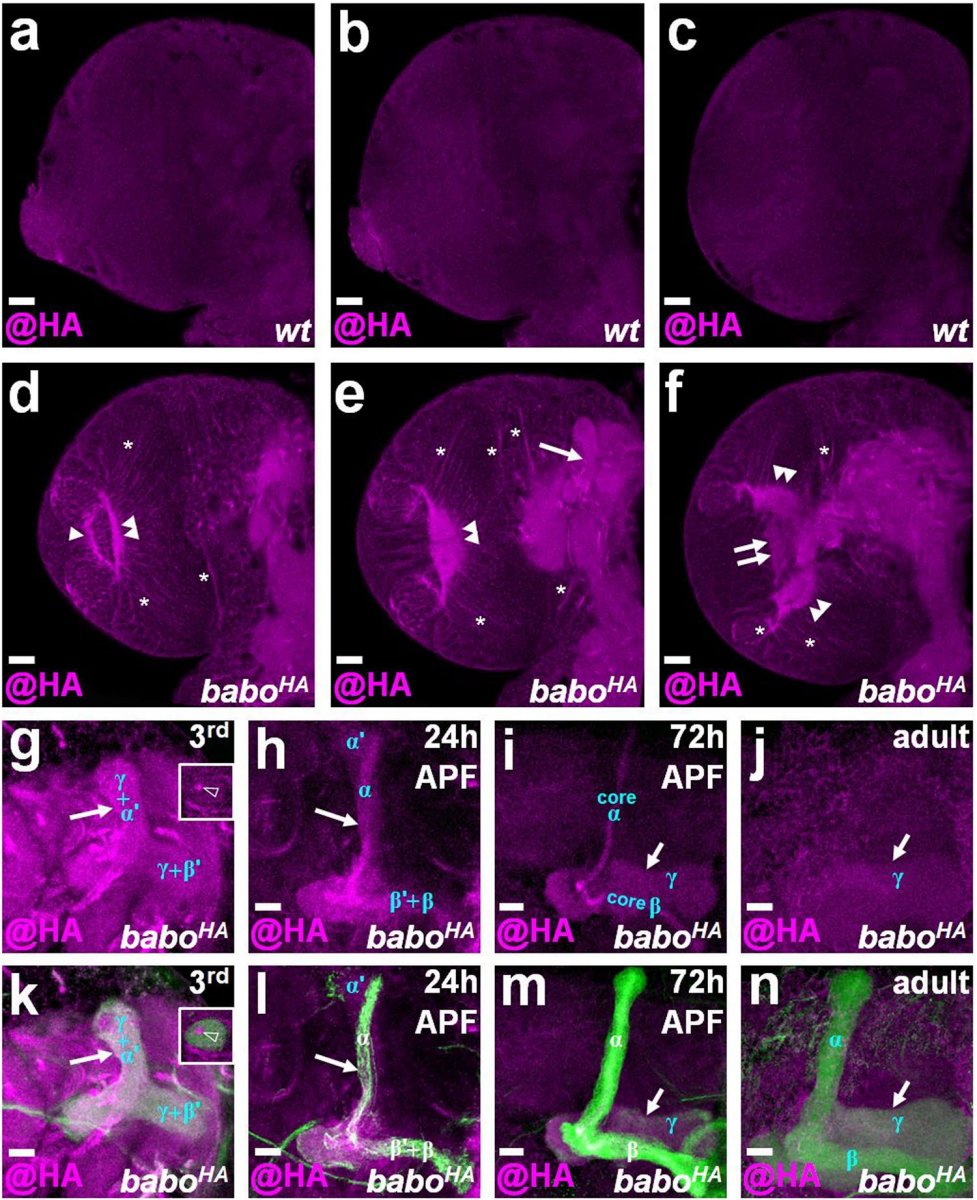
Babo::HA is primarily expressed in nerve fibers and neuropils of the brain. (**a-f**) Three focal planes from anterior to posterior are shown from late third instar larval brains of *wt* (**a-c**) and *babo*^*HA*^ (**d-f**) samples to reveal the endogenous Babo expression pattern. HA staining (magenta) indicates Babo::HA and was clearly observed in nerve fibers (asterisk), lamina (arrowhead), medulla (double arrowhead), lobule (double arrow) and MB lobe (arrow) in the *babo*^*HA*^ sample (**d-f**) but not in the *wt* sample (**a-c**). (**g-n**) Babo::HA expression (magenta) was apparent in the MB lobe (arrow) from the late third instar larval stage (3^rd^) to adulthood. The newly generated MB neurons was indicated in the peduncle section (open arrowhead in insets of **g,k**) and core α and β in (**h,i**). Fasciculin II (shown in green) was used to label the MB lobe position. Scale bar: 20 μm for panels a-f and 10 μm for panels g-n.

After validating the *babo*^*HA*^ knock-in fly, we then determined the Babo expression pattern in the developing brain. In general, we found that Babo was expressed in neuropils and nerve fibers at different developmental stages, ranging from the early larval stage to adulthood (Fig. 3a–f, Supplemental Figs. 1, 2). Since Babo is known to play important roles in the axonal and dendritic morphogenesis of MB neurons in the central brain and Tm neurons in the medulla of the optic lobe^3,5^, we specifically examined Babo::HA expression in MB neurons and neurons within the developing optic lobe. Babo::HA was clearly expressed in the MB lobe from larvae to adults (Fig. 3g–n). We then carefully profiled Babo::HA localization throughout developmental stages. At the late third instar larval stage, we found that the Babo signal appeared in most of the MB lobe with a concentrated signal in the center of the peduncle (Fig. 3g,k), suggesting that Babo may be expressed not only generally in MB γ and α′/β′ neurons but also highly in the newly generated MB neurons. Notably, the Babo::HA expression in MB γ neurons and newly generated MB neurons persisted until the mid-late pupal stage, whereas the Babo::HA expression in MB α′/β′ neurons was greatly reduced at the mid pupal stage (Fig. 3g–i,k–m). At the adult stage, the Babo::HA expression was only observed in MB γ neurons (Fig. 3j,n). In the optic lobe, we found that Babo::HA was initially expressed in inner and outer proliferation centers of the developing optic lobe from early- to mid-larval stages (Supplemental Fig. 1). Correspondingly, Babo::HA expression was later seen in the lamina and medulla neuropils and in the structure that develops into the lobule neuropil at the late third instar larval stage (Fig. 3d–f, Supplemental Fig. 2a–d). Since Babo acts together with type II TGF-β receptors to transduce TGF-β signaling^1^, we wondered whether co-localization of Babo and type II TGF-β receptors can be visualized in the brain. Interestingly, we found that Wit, a type II TGF-β receptor, was generally expressed in neuropils and nerve fibers just like Babo, and Babo::HA and Wit were strongly co-expressed in the developing optic lobe (Supplemental Fig. 3a–f). In addition to its neuronal expression, Babo::HA signal was found to tightly surround glial cell bodies, especially in the optic lobe region (Supplemental Fig. 3g–l), implying the possibility that Babo may also be expressed in glia cells. Taken together, our data using Babo::HA immunostaining to reveal endogenous Babo expression patterns lead us to conclude that Babo is expressed in brain regions enriched with neurites, including the MB, optic lobes and many other neuropils and nerve fibers.

### Utilization of the *babo*^*HA*^ fly to validate *babo* as a miR-34 target gene

**Figure 4:**
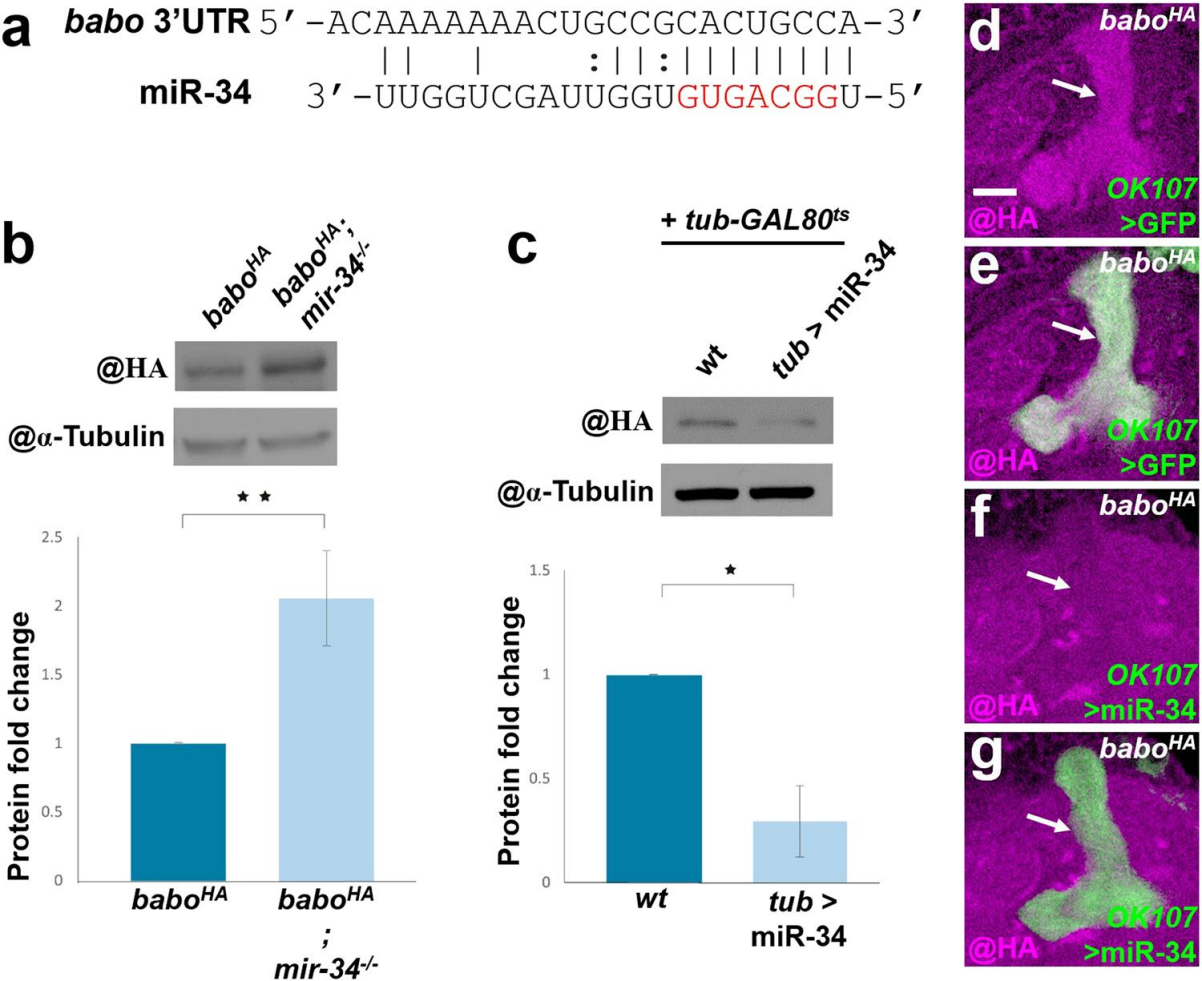
Using the *babo*^*HA*^ fly as a tool to reveal *babo* as a miR-34 target gene. (**a**) The miR-34 seeding sequence was aligned to the *babo* 3'UTR as predicted by TargetScan. (**b,c**) Babo::HA expression was increased in the *mir-34*-deficient adult head and decreased in the miR-34-overexpressing animal, according to western blot analysis. *tub-GAL4* together with temperature-sensitive *tub-GAL80* were used to overexpress miR-34 in (**c**). (**d–g**) Babo::HA expression was substantially reduced in MB lobes (arrows) of the late third instar larval brain, as illustrated with single confocal sections of *babo*^*HA*^ (**d,e**), and *babo*^*HA*^ with *babo-a* RNAi driven by *GAL4-OK107* (**f,g**). HA staining (magenta) and mCD8::GFP signal (green) were used to reveal the Babo::HA expression and the position of MB lobes, respectively. Scale bar: 10 μm for panels d-g.

Our previous study showed that overexpression of miR-34 impairs axon pruning in MB neurons, and interestingly, this axon pruning defect can be rescued by overexpressing Babo^8^. Based on the microRNA target gene prediction algorithm, TargetScan, the 3' untranslated region (UTR) of the *babo* gene contains a putative miR-34 target site (Fig. 4a)^9^. However, the expression level of Babo mRNA was only mildly downregulated (91% remaining) in S2 cells when miR-34 was overexpressed^9^. Therefore, it has been unclear whether *babo* is a true miR-34 target gene in the *Drosophila* brain. To address this question, we quantified Babo::HA expression after modulating miR-34 in the *babo*^*HA*^ fly. We found that the Babo::HA expression was upregulated (207 ± 34% of control levels, *p* < 0.01) in the *mir-34*-deficient adult head and downregulated (30 ± 17% remaining, *p* < 0.05) in miR-34 overexpressing animals, according to western blot analysis (Fig. 4b,c). Thus, we conclude that miR-34 negatively regulates Babo expression. Notably, we further observed that Babo::HA expression was significantly reduced in the MB lobe when miR-34 was overexpressed using *GAL-OK107* (Fig. 4d,e), and the knockdown efficiency was comparable to that produced by expression of *babo-a* RNAi under control of the same driver (Supplemental Fig. 4). These findings support the idea that the miR-34-induced MB axon pruning defect in our previous study was probably due to downregulation of the Babo expression^8^. Taken together, our data suggest that *babo* is a miR-34 target gene, which participates in crucial biological processes such as the axon pruning of MB neurons. Furthermore, our results demonstrate the utility of the *babo*^*HA*^ fly for monitoring Babo expression in the brain.

## Discussion

Babo acts as an important regulator of diverse and complicated developmental processes, such as neuroblast proliferation in brain development and neuronal morphogenesis in neural circuit formation^3–5^. However, the Babo expression pattern in brain has remained enigmatic due to difficulties in detecting the endogenous protein. In this study, we generated a *babo*^*HA*^ knock-in fly to visualize the expression pattern of endogenous Babo protein. Babo::HA was expressed in brain structures enriched with neurites, including the MB and optic lobes (Figs. 2, 3). Interestingly, Babo::HA and Wit (a type II TGF-β receptor) displayed similar neuropil expression patterns in the late third instar larval brain, especially in the optic lobe region (Supplemental Fig. 3a–f). These results correspond nicely to previous functional studies that suggested Babo and Wit can form a regulatory complex, which is crucial for axon pruning of MB neurons and dendritic patterning of Tm neurons in the optic lobe^3,5^. We also utilized the *babo*^*HA*^ fly to solve an existing puzzle of whether *babo* is truly a miR-34 target gene. We found that *mir-34* depletion upregulated Babo::HA, while miR-34 overexpression downregulated its expression (Fig. 4). Therefore, we are confident in our conclusion that *babo* is indeed a miR-34 target gene. These results suggest that the *babo*^*HA*^ fly can serve as an excellent reagent in studies that seek to clarify the participation of Babo in the brain development and formation of functional neural circuits. Despite our elucidation of the endogenous Babo expression pattern in the brain, at least three issues remain to be addressed. First, we cannot distinguish the expression patterns of each Babo protein isoform since the HA tag was inserted immediately upstream of the common amino acid stop codon for Babo-A, -B and -C. Second, despite our finding that Babo tightly encases glia cell bodies (Supplemental Fig. 3g–l), we still cannot be entirely sure whether Babo is expressed in glia. Third, we also did not specifically assess whether Babo expression is present in tissues other than the brain. In the future, RNAi reagents specific to certain Babo variants may be used to knock down expression in neurons and/or glia, followed by assessment of Babo expression patterns. Additionally, expression in other tissues of the *babo*^*HA*^ fly can be tracked to provide a complete description of the distribution of endogenous Babo during development. Such studies may hint at unexplored territories and functions of Babo in the animal.

## Methods

### Generation of the *babo*^*HA*^ knock-in fly

Two PCR DNA fragments carrying a HA-tag in frame with the C-terminus of Babo immediately upstream of the translational stop codon were generated with two pairs of primers (Fig. 2a): (L-arm primers) atccactagtgctagcttatcggaaagggacgtttcggcgaggtctggcgt and taatctggaacgtcatatggatag ttcttgaccttgtcctccacactgatgctagcaa; (R-arm primers) tgacgttccagattacgcttaattgtgcttcagtttgacgagtagccc tcgtgtcac and tagatgcatgctcgagaacaggatttataaagggatatgttcacaggcaat. L-arm and R-arm PCR products were cloned into the SpeI/XhoI sites of pCR2-TOPO vector with an In-Fusion HD cloning Kit (Clontech) to generate the pCR2-TOPO-Babo-1xHA-stop HR donor vector. The CRISPR target site of the *babo* gene for CRISPR/Cas9 recognition was designed with the flyCRISPR optimal target finder platform (https://flycrispr.org/target-finder/)^14^. The sequence of a target site near the stop codon of the *babo* gene was selected (acaaggtcaagaactgattg; Fig. 2a). The target site (guide RNA) fragment was generated by annealing primers with the sequences: cttcgacaaggtcaagaactgattg and aaaccaatcagttcttgaccttgtc. The annealed target site fragment was cloned into the BbsI site of pBFv-U6.3 to generate the pBFv-U6.3-Babo-stop gRNA plasmid^10,11^. The pCR2-TOPO-Babo- 1xHA-stop HR donor vector and the pBFv-U6.3-Babo-stop gRNA plasmid were injected into a Cas9 founder strain by WellGenetics Inc. to generate the *babo*^*HA*^ knock-in fly.

### Fly strains

The following fly strains were used in this study: (1) *UAS-mCD8::GFP*^15^; (2) *da-GAL4* (Bloomington stock [BL] 55850); (3) *tub-GAL4*^15^; (4) *elav-GAL4*^15^; (5) *GAL4-OK107*^8^; (6) *UAS-babo-a RNAi* (BL 44400)^6^; (7) *UAS-mir-34*^8^; (8) *UAS-Babo-A*^5^; (9) *tub-GAL80*^*ts*^ (BL7017); (10) *w*^1118^ (BL5905); (11) *mir-34*^−/−^^15^.

### Fly brain preparation, image processing and western blot analysis

Dissection, immunostaining and mounting of fly brains were performed as described previously^15^. Primary antibodies used in immunostaining were rabbit anti-GFP (1:800, Life Technologies), rat anti-HA (1:500, Roche), mouse anti-Fasciculin II (1D4), anti-Repo (8D12), and anti-Wit (23C7) (1:50, Developmental Studies Hybridoma Bank). Secondary antibodies conjugated to Alexa 488, 546 or 647 (1:800) were purchased from Life Technologies. Immunofluorescence images were captured using a Zeiss LSM 700 confocal microscope and were processed using the Zeiss LSM image browser to project images from confocal stacks and Photoshop (Adobe) to adjust image intensity. No other image processing was performed. The western blots were conducted using Bio-Rad mini-PROTEAN tetra and Sema-dry transfer systems. Primary antibodies used for western blotting were rabbit anti-Babo (1:500; Abcam #14681 and #14682; MyBioSource #540193, #540486 and #610062), rabbit anti-HA (1:1,000, Cell Signaling), rat anti-HA (1:5,000, Roche), mouse anti-β-Actin (1:5,000, Millipore), mouse anti-α-Tubulin (1:5,000, GeneTex). Secondary antibodies conjugated to Horseradish peroxidase (1:100,00) were purchased from The Jackson Laboratory. The western blot signals were detected using GE Healthcare ECL reagent and Fuji X-ray films. The quantitation of protein bands was performed with ImageJ, and Student’s t-test was used for statistical analysis. Quantitation of immunostaining was also performed with ImageJ, and one-way ANOVA with post hoc Tukey test was used for statistical analysis.

## Supplementary information

Supplemental figures and table.
