# Supplementary material for: Visualization of Endogenous Type I TGF-β Receptor Baboon in the Drosophila Brain
Source: Sci Rep. 2020 Mar 20;10:5132. doi: 10.1038/s41598-020-61950-y (PMC7083856; doi:10.1038/s41598-020-61950-y)
Supplement: Supplementary file 1 — Supplemental figures and table. [file 41598_2020_61950_MOESM1_ESM.docx]

**Visualization of Endogenous Type I TGF-β Receptor Baboon in the *Drosophila* Brain**

Yen-Wei Lai^1,2,3^, Sao-Yu Chu^1,2,3^, Jian-Chiuan Li^2^, Po-Lin Chen^2,3^, Chun-Hong Chen^2,3^* and Hung-Hsiang Yu^1^*

^1^Institute of Cellular and Organismic Biology, Academia Sinica, Taipei, Taiwan.

^2^National Institute of Infectious Diseases and Vaccinology, National Health Research Institutes, Miaoli County, Taiwan.

^3^Institute of Molecular and Cellular Biology, College of Life Science, National Taiwan University, Taipei, Taiwan.

*Corresponding authors

E-mail: chunhong@nhri.org.tw and [samhhyu@gate.sinica.edu.tw](mailto:samhhyu@gate.sinica.edu.tw)

**Supplemental figure 1**

**
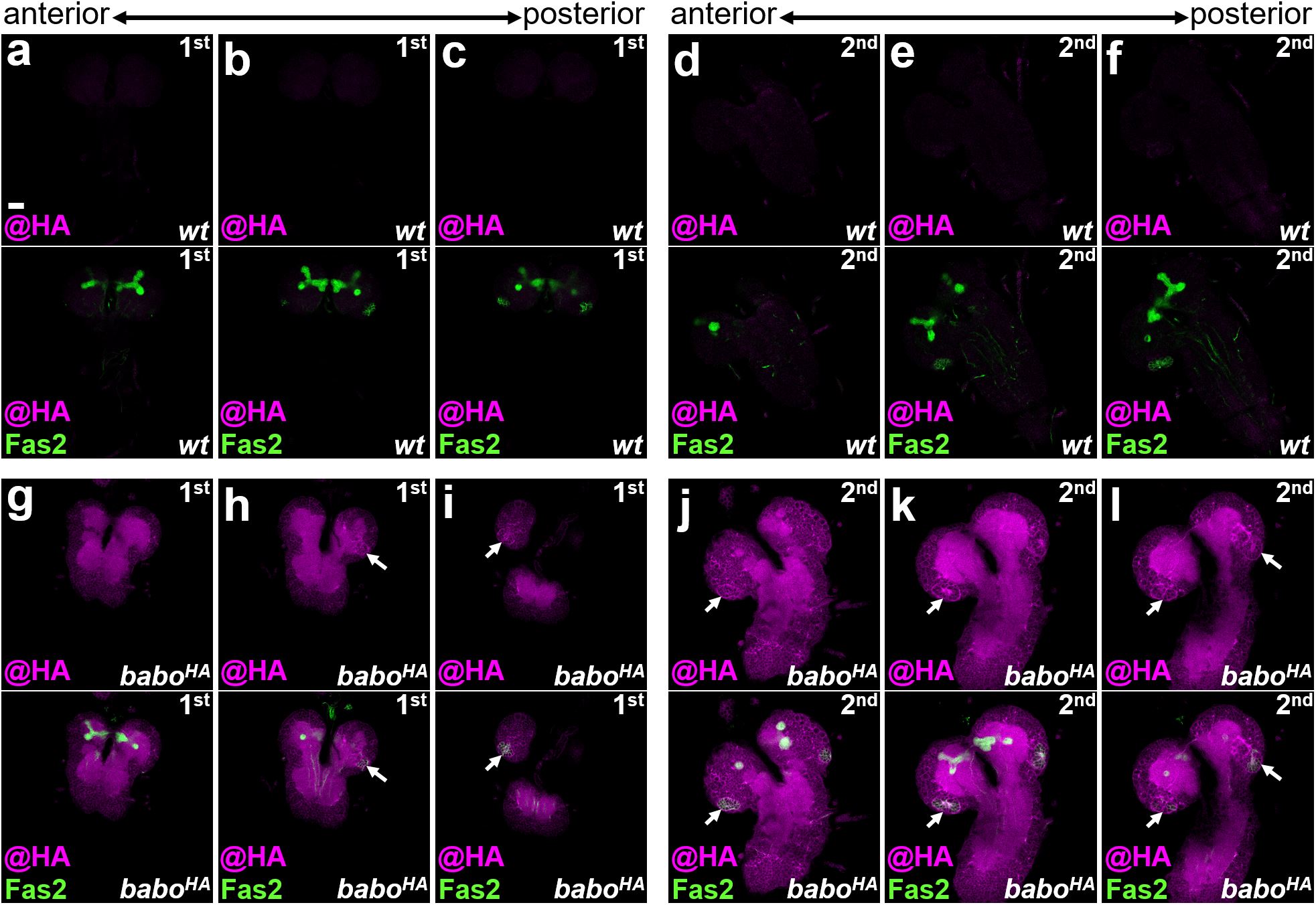
**

**Supplemental Figure 1. The Babo::HA expression pattern in the early- and mid-larval brains**.

(a-l) Three focal planes of *wt* (a-f) and *babo^HA^* (g-l) samples from anterior to posterior were examined to assess the endogenous Babo expression patterns in first (a-c, g-i) and second (d-f, j-l) instar larval brains. Babo::HA (indicated by HA staining, shown in magenta) and Fasciculin II (Fas2; shown in green). Inner and outer proliferation centers of the developing optic lobe are indicated by arrows. Scale bar: 20 μm.

**Supplemental figure 2**

**
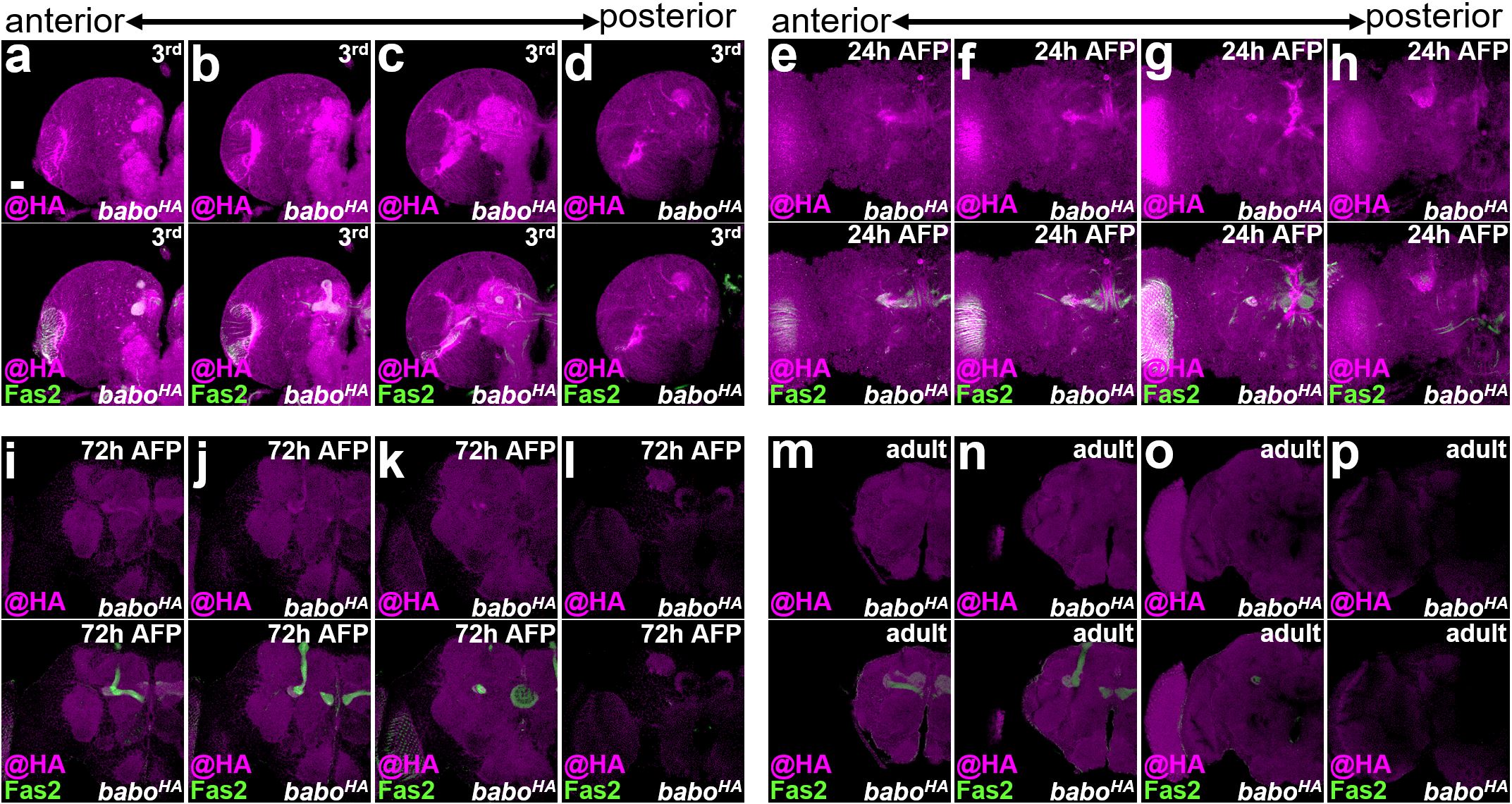
**

**Supplemental Figure 2. The Babo::HA expression pattern in the late larval, pupal and adult brains**.

(a-p) Four focal planes of *babo^HA^* samples (a-p) from anterior to posterior were examined to reveal the endogenous Babo expression patterns in late third instar (a-d), 24 h after puparium formation (APF, e-h), 72 h APF (i-l) and adult (m-p) brains. Babo::HA (indicated by HA staining, shown in magenta) and Fasciculin II (Fas2; shown in green). Scale bar: 20 μm.

**Supplemental figure 3**

**
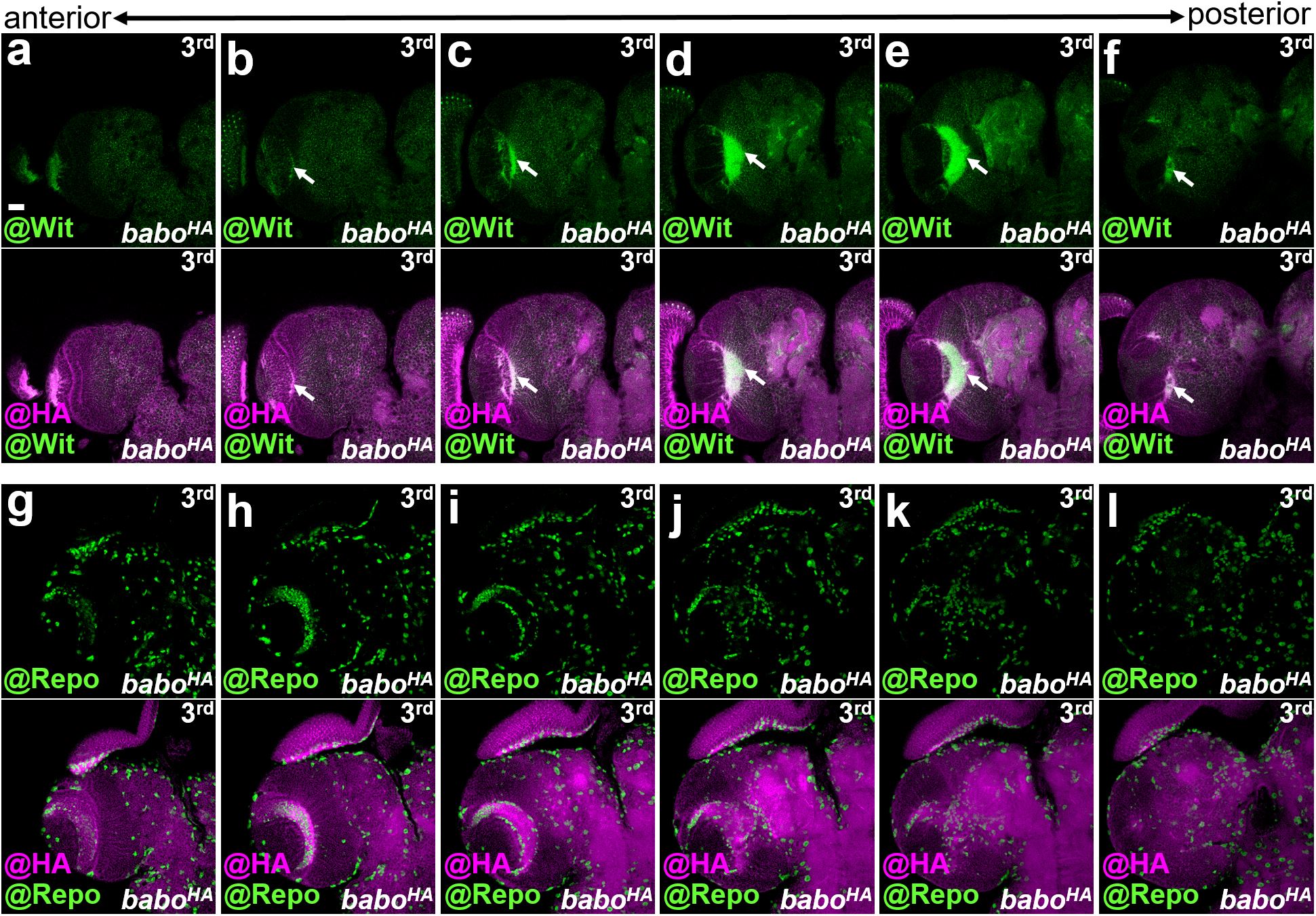
**

**Supplemental Figure 3. Babo::HA, Wit and Repo expression patterns in the brain**.

(a-l) Six focal planes of *babo^HA^* samples (a-p) from anterior to posterior were examined to reveal the endogenous Babo (a-p), Wit (a-f) and Repo (g-l) expression patterns in late third instar brains. Babo::HA (indicated by HA staining, shown in magenta), Wit (a type II TGF-β receptor, shown in green in panels a-f), and Repo (a glial cell marker, shown in green in panels g-l). Babo::HA and Wit were strongly co-expressed in the optic lobe region (arrows). Scale bar: 20 μm.

**Supplemental figure 4**

**
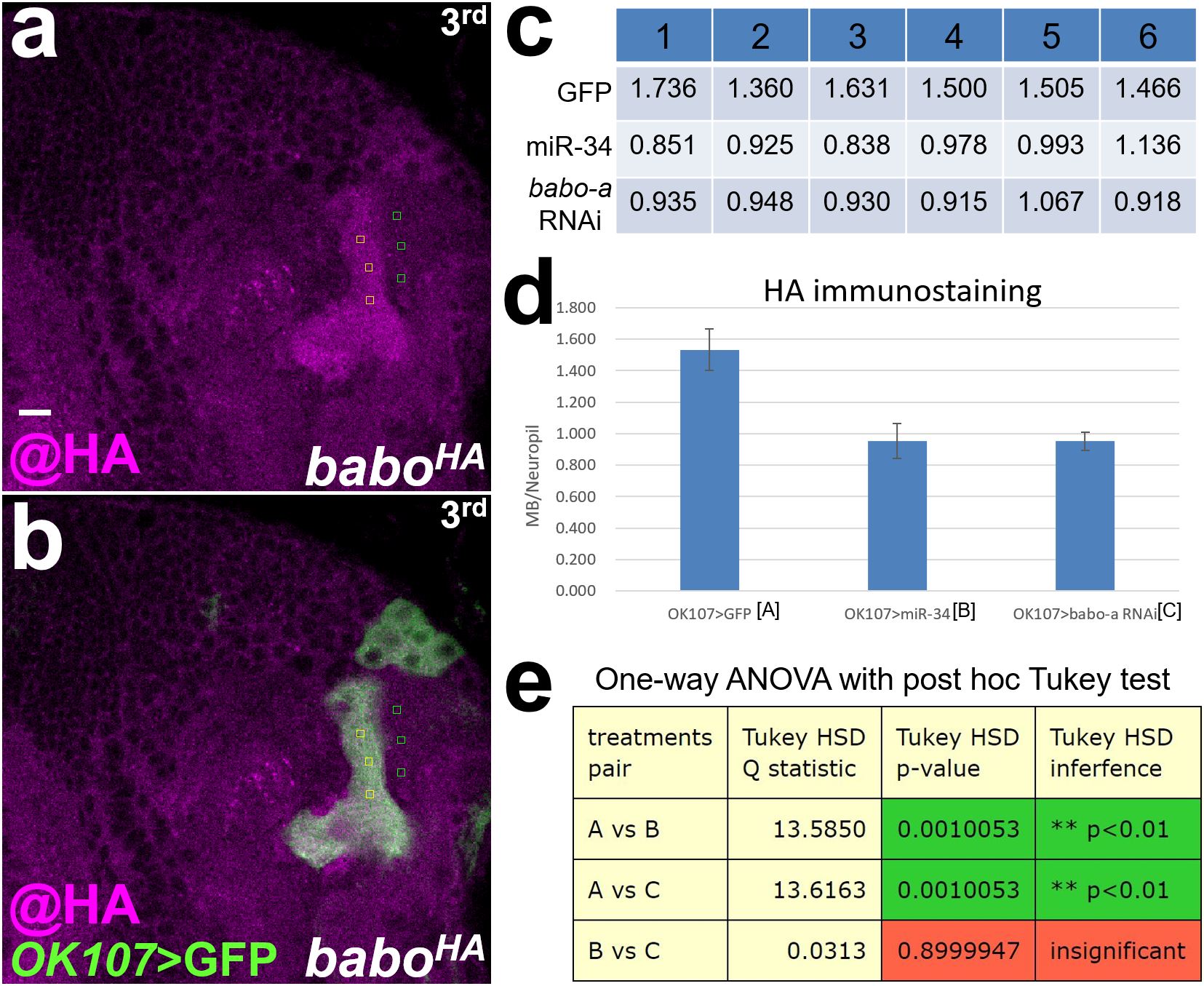
**

**Supplemental Figure 4. Knockdown efficiency of miR-34 and *babo-a* RNAi in MB lobes**.

(a-b) To compare the Babo knockdown efficiencies after expression of miR-34 and *babo-a* RNAi in MB neurons, we measured pixel intensity in three rectangular regions in the MB lobe (yellow; Babo knockdown area) and the nearby neuropil (green; an area where Babo is not knocked down) from a single focal section of late third instar larval *babo^HA^* brain. Pixel intensities of yellow regions were normalized to those of green regions to obtain the numbers shown in panel c. Images from Figure 2d and 2e are used to illustrate the measurement of Babo knockdown efficiency. (c) The measurements were made in six independent *babo^HA^* MB lobe samples from GFP controls [A], and flies with miR-34 overexpression [B] and *babo-a* RNAi knockdown [C] driven by *GAL4-OK107*. (d-e) The average and statistical test results (one-way ANOVA with post hoc Tukey test) for GFP control, miR-34 overexpression and *babo-a* RNAi knockdown are shown in panels d and e. miR-34 overexpression and *babo-a* RNAi knockdown generate similar Babo knockdown efficiencies in MB neurons. Scale bar: 10 μm.

**Supplemental Table 1. Genotypes of the flies in the figures**

| **Figure** | **Genotype** |
| --- | --- |
| 1a, 1c-lane1, 2b-lane1, 3a-c, S1a-f | *w^1118^;+;+;+* |
| 1b-lane1 | *elav-GAL4/w;+;+;+* |
| 1b-lane2 | *elav-GAL4/UAS-Babo-A;+;+;+* |
| 1b-lane3 | *elav-GAL4/w;UAS-babo-a RNAi/+;+;+* |
| 1c-lane2 | *UAS-Babo-A/w;+;da-GAL4/+* |
| 1c-lane3 | *UAS-Babo-A/w;+;tub-GAL4/+* |
| 2b-lane2, 3d-n, 4b-lane1, S1g-l, S2, S3 | *w;babo^HA^;+;+* |
| 2c-lane1 | *elav-GAL4/w;babo^HA^/+;+;+* |
| 2c-lane2 | *elav-GAL4/w;babo^HA^/UAS-babo-a RNAi;+;+* |
| 2d, 2e, S4a-b | *w;UAS-mCD8::GFP/+;+;GAL4-OK107/+* |
| 2f, 2g, 4d, 4e | *w;babo^HA^/+;UAS-mCD8::GFP/+;GAL4-OK107/+* |
| 2h, 2i | *w;babo^HA^/UAS-babo-a RNAi;UAS-mCD8::GFP/+;GAL4-OK107/+* |
| 4b-lane2 | *w;babo^HA^;mir-34^-/-^;+* |
| 4c-lane1 | *w,UAS-miR-34/+;tub-GAL80^ts^/+;+* |
| 4c-lane2 | *w,UAS-miR-34/+;tub-GAL80^ts^/tub-GAL4;+* |
| 4f, 4g | *w;babo^HA^/UAS-miR-34;UAS-mCD8::GFP/+;GAL4-OK107/+* |
